# Diagnostic performance of clinical photon-counting CT in the assessment of peri-implantitis – an ex vivo diagnostic accuracy study

**DOI:** 10.1186/s40729-026-00722-1

**Published:** 2026-10-28

**Authors:** Maurice Ruetters, Christian Mertens, Anna Felten, Blagovest Blagoev, Alexander-Nicolaus Spies, Ti-Sun Kim, Heinz-Peter Schlemmer, Stefan Schoenberg, Matthias Froelich, Marc Kachelrieß, Stefan Sawall

**Affiliations:** 1 Department for Conservative Dentistry, Medical Faculty Heidelberg, Heidelberg University, University Hospital Heidelberg , Im Neuenheimer Feld 400, 69120 Heidelberg, Germany; 2 Department of Oral- and Maxillofacial Surgery, Heidelberg University, University Hospital Heidelberg, Im Neuenheimer Feld 400, 69120 Heidelberg, Germany; 3 Department of Radiology, German Cancer Research Center (DKFZ), Im Neuenheimer Feld 280, 69120 Heidelberg, Germany; 4 Department of Clinical Radiology and Nuclear Medicine, University Hospital Mannheim, Theodor-Kurz-Ufer 1-3, 68167 Mannheim, Germany; 5 Medical Faculty, Heidelberg University, Im Neuenheimer Feld 672, 69120 Heidelberg, Germany; 6 Division of X-Ray Imaging and CT, German Cancer Research Center (DKFZ), Im Neuenheimer Feld 280, 69120 Heidelberg, Germany

**Keywords:** Peri-implantitis, Photon-counting CT, CBCT, 3D dental imaging, Implant diagnostics, Image quality

## Abstract

**Purpose:**

To evaluate the diagnostic performance of photon-counting computed tomography (PCCT) for the assessment of peri-implant bone defects and to compare it with cone-beam computed tomography (CBCT) at comparable radiation doses.

**Methods:**

Circular peri-implant defects were artificially created around 27 titanium implants placed in nine porcine mandibles. Defect morphology was digitized using an intraoral scanner (STL, stereolithography file format) as reference standard. Specimens were imaged with high-resolution CBCT (HD-CBCT) and PCCT at clinically commonly used dose levels, which were nearly identical for both modalities to ensure comparability. Vertical defect depth and horizontal defect width were measured at predefined locations by two calibrated readers. Agreement with the reference standard was assessed using paired statistical testing, Bland-Altman analysis, mean absolute error (MAE), relative error metrics, and intraclass correlation coefficients (ICC).

**Results:**

For vertical defect depth (mean reference 4.68 ± 2.20 mm), PCCT demonstrated significantly lower MAE than CBCT (0.14 mm vs. 0.67 mm; *p* < 0.001) and narrower limits of agreement. Relative absolute error was 4.3% for PCCT and 17.4% for CBCT. For horizontal defect width (mean reference 0.99 ± 0.39 mm), both modalities slightly underestimated the defect width compared to the reference standard of the oral scanner. However, PCCT showed lower MAE (0.077 mm vs. 0.18 mm; *p* < 0.001) and reduced proportional variability. Intra- and inter-reader reliability were consistently higher for PCCT (ICC up to 0.994) compared with CBCT.

**Conclusions:**

Within the limitations of this ex vivo model, PCCT provided more accurate and reproducible three-dimensional measurements of standardized peri-implant bone defects than CBCT. These findings need to be confirmed by further in vivo studies to determine its clinical diagnostic value and potential impact on treatment planning.

**Graphical abstract:**

**Figure:**
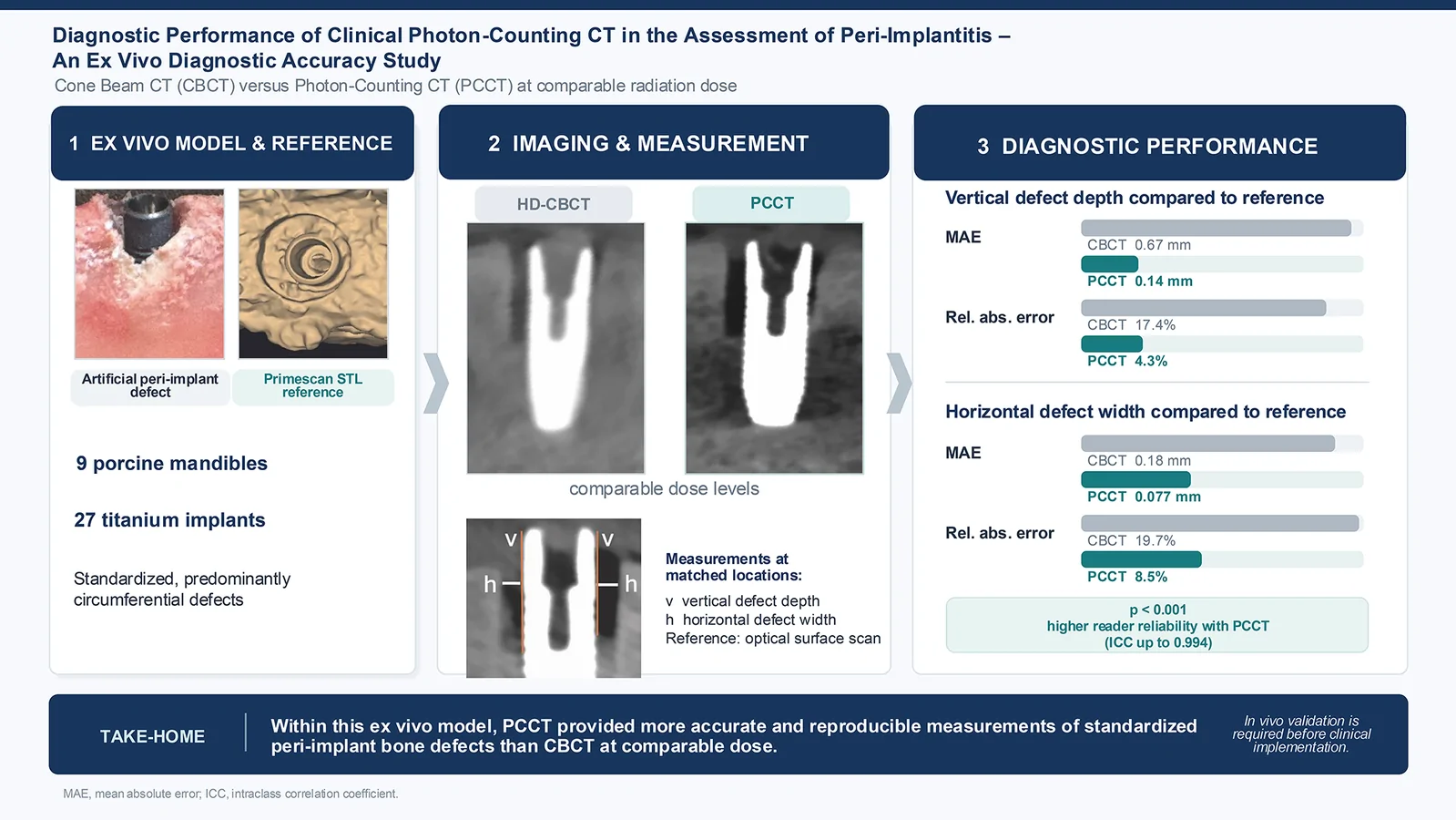

## Summary Box

What is known:

What this study adds:

• CBCT is widely used for characterizing peri-implantitis but is limited by metal arti-facts.

• PCCT offers a three-dimensional imaging alternative that might reduce metal arti-facts.

• This is the first ex vivo diagnostic accuracy study comparing CBCT and PCCT in imag-ing of peri-implant bone defects.

• PCCT allows for a superior visualization of vertical and horizontal peri-implant bone defects compared to CBCT.

## Background

Peri-implantitis is a pathological inflammatory condition affecting the peri-implant tissues, characterized by progressive bone loss around dental implants [1, 2]. The exact histopathological and clinical mechanisms leading to the transition from peri-implant mucositis to peri-implantitis remain incompletely understood. Peri-implant infections are characterized by a high prevalence. According to the literature, the weighted mean prevalence is 43% (confidence interval: 32–54%) for peri-implant mucositis (1,196 patients, 4,209 implants) and 22% (confidence interval: 14–30%) for peri-implantitis (2,131 patients, 8,893 implants) [3]. The disease can manifest early after implantation and follows a non-linear, often accelerating progression [2]. Clinically, peri-implant defects are characterized by increased signs of inflammation and deeper probing depths compared to baseline measurements [1, 2]. Histologically, peri-implantitis lesions exhibit larger inflammatory areas than periodontal defects. Surgical exploration frequently reveals a circumferential pattern of bone loss [4, 5]. Major risk factors for the development of peri-implantitis include a history of chronic periodontitis, inadequate plaque control, and lack of maintenance care after implantation [6–8]. In addition to microbial factors, patient-specific risk factors such as poor oral hygiene and systemic diseases play a crucial role [8, 9]. Evidence also suggests that additional factors such as submucosal residual cement, insufficient keratinized peri-implant mucosa, and unfavorable implant positioning may contribute to disease progression [10]. Morphologically, peri-implant bone defects have been classified by Schwarz et al., allowing for a systematic categorization based on their three-dimensional structure [11]. This differentiation is crucial for determining the appropriate therapeutic approach. The radiological assessment of peri-implant defects plays a key role in detecting and evaluating bone loss around implants. Currently, conventional radiographic techniques, cone-beam computed tomography (CBCT), and computed tomography (CT) are the primary imaging modalities used to assess peri-implant bone structures. Intraoral radiographs provide a simple and cost-effective evaluation of marginal bone levels but are limited by their two-dimensional nature and inability to reliably depict buccal or lingual defects [12]. Panoramic radiographs offer a broader overview but suffer from lower resolution and increased distortion compared to intraoral images, making them less suitable for diagnosing small peri-implant defects. CBCT represents a significant advancement in imaging, as it enables three-dimensional visualization of peri-implant bone structures [12]. However, CBCT scans are affected by metal artifacts, particularly from implants, which can compromise the accurate assessment of peri-implant defects [13]. Computed tomography provides improved soft tissue visualization but is also affected by metal artifacts and exposes patients to a higher radiation dose compared to CBCT [14]. A promising advancement in imaging technology is photon-counting computed tomography (PCCT). PCCT addresses several limitations of traditional imaging techniques by delivering superior spatial and contrast resolution, effectively reducing metal artifacts, and enabling spectral imaging [15]. Unlike conventional CT systems, PCCT detectors count individual X-ray photons and classify them according to their energy, which enhances material differentiation [15]. Studies have already shown highly promising results regarding PCCT’s ability to visualize dental structures, pathologies and the surrounding bone of teeth and implants [16–19]. Therefore, technology holds particular promise for the early diagnosis of peri-implant conditions such as bone resorption and peri-implantitis. Furthermore, PCCT’s advanced spectral imaging capabilities facilitate more accurate tissue differentiation and a more detailed analysis of material composition. To date, however, no study has specifically evaluated the suitability of PCCT for the characterization of peri-implant osseous defects.

Therefore, the objective of this study is to evaluate the diagnostic potential of PCCT for the detailed assessment of peri-implant bone defects and to compare with existing imaging modalities. The hypothesis is that PCCT can depict peri-implant defects more accurately than CBCT at the same radiation dose.

## Results

### Vertical defect depth

**Fig. 1:**
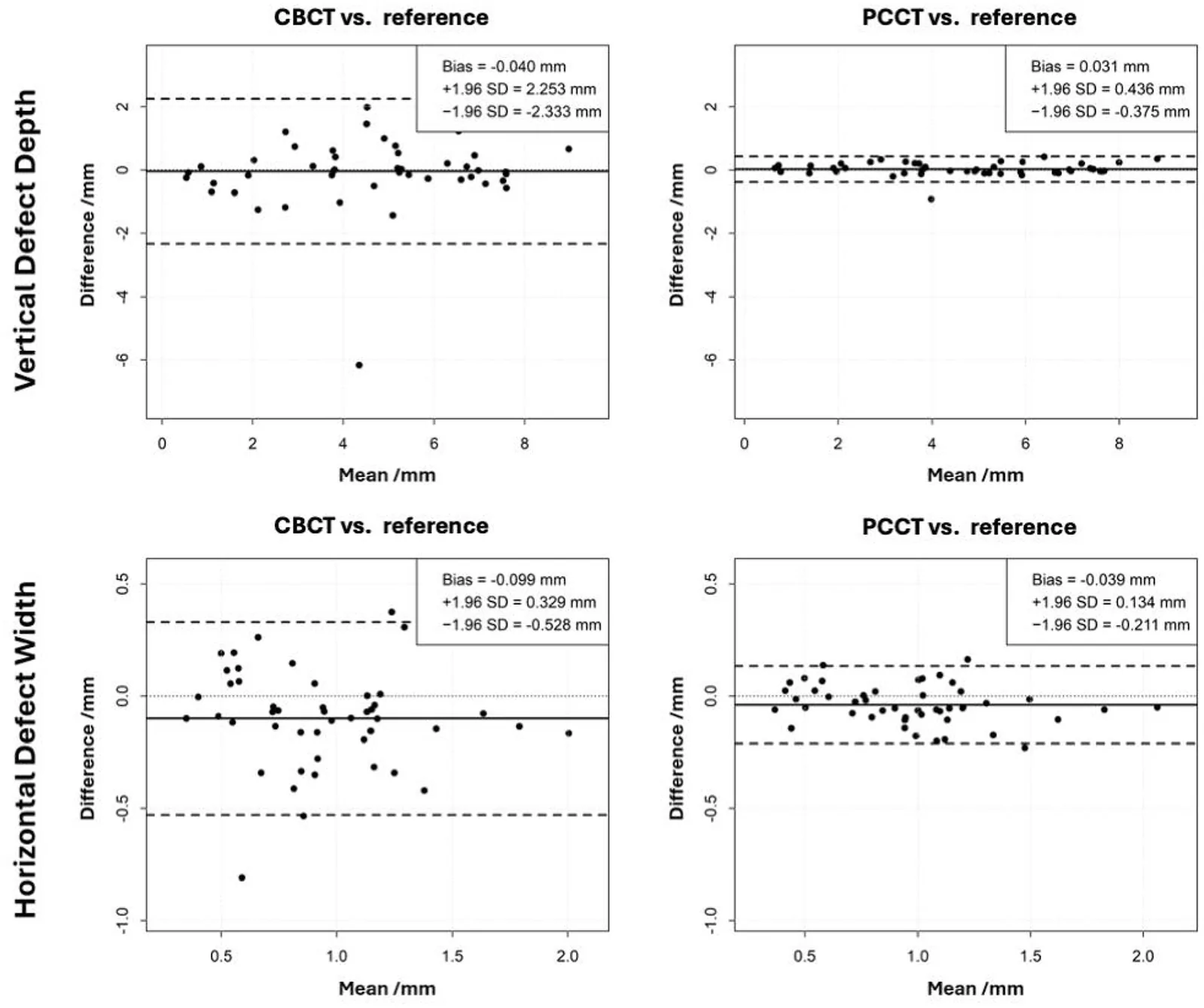
Bland-altman plots of absolute differences between CBCT (left) and PCCT (right) and the reference standard for vertical defect depth (top) and horizontal defect width (bottom). CBCT is shown on the left and PCCT on the right

**Fig. 2:**
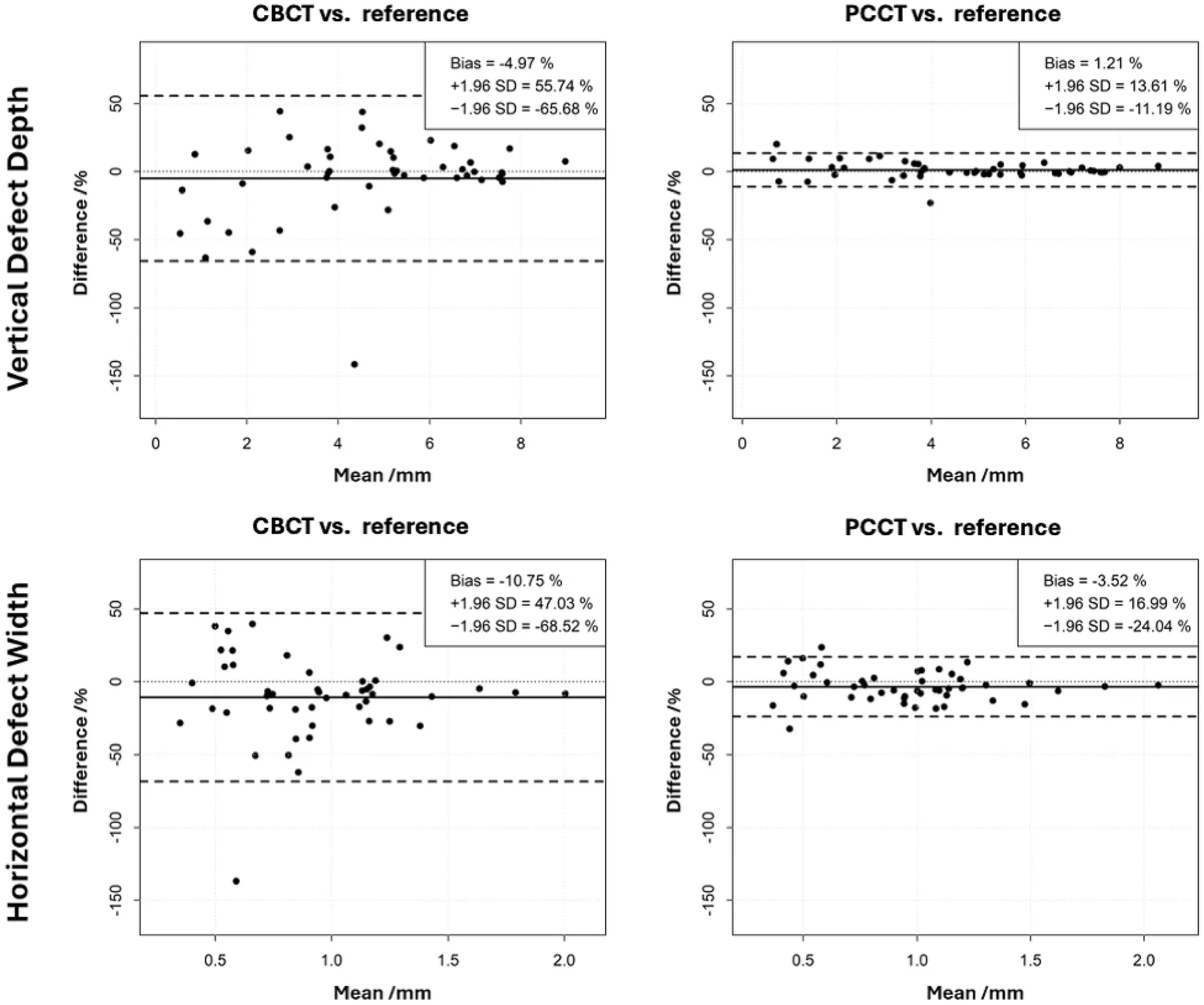
Bland-altman plots of relative differences between CBCT (left) and PCCT (right) and the reference standard for vertical defect depth (top) and horizontal defect width (bottom). CBCT is shown on the left and PCCT on the right

For the vertical defect depth, the mean reference value was 4.68 ± 2.20 mm (range 0.62–8.64 mm). Neither CBCT nor PCCT demonstrated a significant systematic bias relative to the reference standard. Despite the absence of systematic bias, substantial differences in precision were observed. CBCT demonstrated a mean absolute error of 0.67 mm, with 95% limits of agreement ranging from − 2.33 mm to + 2.25 mm. In contrast, PCCT showed a considerably lower mean absolute error of 0.14 mm and narrower limits of agreement from − 0.37 mm to + 0.44 mm. Relative analysis confirmed these findings. The mean relative absolute error was 17.4% for CBCT and 4.3% for PCCT. Percent Bland-Altman analysis demonstrated wide proportional limits of agreement for CBCT (− 66% to + 56%), whereas PCCT showed substantially narrower limits (− 11% to + 14%) (Fig. 1 and 2). Both absolute and relative error comparisons demonstrated significantly lower errors for PCCT compared with CBCT (*p* < 0.001). Intra-reader reliability for Reader 1 was excellent for both modalities (ICC = 0.967 for CBCT and 0.986 for PCCT). Inter-reader reliability was also high, with ICC values of 0.949 for CBCT and 0.994 for PCCT.

### Horizontal defect width

For the horizontal defect width, the mean reference value was 0.99 ± 0.39 mm (range 0.40–2.09 mm). Both CBCT and PCCT demonstrated a small but statistically significant underestimation relative to the reference standard (− 0.10 mm for CBCT and − 0.04 mm for PCCT). CBCT yielded a mean absolute error of 0.18 mm, with limits of agreement ranging from − 0.53 mm to + 0.33 mm. PCCT demonstrated a lower mean absolute error of 0.077 mm and narrower limits of agreement from − 0.21 mm to + 0.13 mm. The mean relative absolute error was 19.7% for CBCT and 8.5% for PCCT. Percent Bland-Altman limits ranged from − 69% to + 47% for CBCT, compared with − 24% to + 17% for PCCT (Fig. 1 and 2). Absolute and relative error comparisons confirmed significantly lower measurement error for PCCT (*p* < 0.001). Intra-reader reliability was good for CBCT (ICC = 0.87) and excellent for PCCT (ICC = 0.98). Inter-reader reliability was 0.883 for CBCT and 0.973 for PCCT.

Across both evaluated distance measures, PCCT demonstrated lower mean absolute errors and narrower limits of agreement compared with CBCT. Relative error analysis showed consistently smaller proportional deviations from the reference standard for PCCT. In addition, intra- and inter-reader intraclass correlation coefficients were higher for PCCT than for CBCT. These findings were consistent across measures of different absolute magnitudes.

## Discussion and conclusion

The present study demonstrates the superiority of PCCT over CBCT in the visualization of peri-implant bone defects in an ex-vivo setting. It provides more accurate and reproducible linear measurements than cone-beam computed tomography (CBCT). While systematic bias was minimal or absent for most measurements, substantial differences in measurement precision were observed between modalities. For the vertical defect depth, both modalities showed no significant systematic bias relative to the reference standard obtained using an intra-oral scanner. However, CBCT exhibited considerably wider limits of agreement and higher mean absolute errors compared with PCCT. For the horizontal defect width, both modalities demonstrated a small but statistically significant underestimation of the reference standard, although the magnitude of this bias was small in absolute terms. Again, PCCT showed narrower limits of agreement and lower absolute errors. Importantly, the superiority of PCCT remained evident after normalization for defect size. Relative error analysis demonstrated consistently lower proportional deviations for PCCT across both defect types. Moreover, percent Bland-Altman analysis revealed substantially narrower proportional limits of agreement for PCCT. These findings indicate that the observed differences are not solely attributable to scaling effects related to the magnitude of the measured structures, but rather reflect intrinsic differences in measurement performance between the imaging modalities. The consistently higher intra- and inter-reader intraclass correlation coefficients observed for PCCT further support its superior reproducibility. While CBCT also demonstrated generally good reliability, reproducibility was lower and more variable across defect types. The higher reproducibility of PCCT may be explained by the overall improved image stability achieved with photon-counting CT under the applied acquisition conditions. The combination of higher tube voltage and tin prefiltration results in a spectrally hardened X-ray beam, which enhances penetration of high-attenuation materials and reduces beam hardening effects in the vicinity of metallic implants. In addition, clinical CT systems generally exhibit lower scatter radiation compared with CBCT systems due to differences in acquisition geometry and detector configuration and due to advanced scatter correction algorithms, which may further contribute to more stable attenuation measurements. Together with the reduced electronic noise inherent to photon-counting detectors, these factors likely lead to decreased artifact expression and more consistent delineation of peri-implant structures, thereby improving measurement reproducibility [20–22].

The finding that relative errors for CBCT were comparable across defects of different sizes suggests that measurement variability in CBCT may scale proportionally with structure size. In contrast, PCCT demonstrated lower proportional variability, indicating greater robustness across a range of measurement magnitudes. The results are in line with earlier studies, also highlighting the superiority of PCCT compared to CBCT for several dental indications [16–19].

From a clinical perspective, the findings of this ex vivo study suggest that the improved visualization of peri-implant defect morphology provided by PCCT may have potential relevance for diagnostic assessment and treatment planning. However, as the present study was performed under standardized ex vivo conditions, no conclusions regarding clinical decision-making, prognostic assessment, surgical planning, or patient outcomes can be drawn. These potential benefits therefore require confirmation in prospective in vivo studies. Independent feasibility studies have additionally suggested that head and neck PCCT examinations may allow opportunistic assessment of dental and peri-implant structures [19, 23]. This approach may allow previously undetected pathologies to be diagnosed and treated at an earlier stage, thereby potentially preventing further disease progression. However, this specific application was not investigated in the present study.

This study has several limitations. The ex vivo porcine model does not fully reproduce clinical imaging conditions, as surrounding soft tissues, patient motion, and inflammatory tissue components were absent. Consequently, image noise and attenuation characteristics may differ from in vivo situations. In addition, only standardized, predominantly circumferential peri-implant defects were investigated. This controlled defect design may facilitated reproducible measurements and direct comparison between imaging modalities but does not represent the full morphological spectrum observed clinically. Peri-implant bone loss may present as irregular, non-circumferential, dehiscence-type, crater-like, horizontal, vertical, or combined defect configurations. Diagnostic performance may vary depending on defect geometry, the number and thickness of remaining bony walls, defect angulation, and defect dimensions. Therefore, the present results cannot necessarily be extrapolated to all clinically occurring peri-implant defect morphologies. Future studies should include a broader range of clinically representative defect configurations. Another potential source of measurement uncertainty is the intraoral scanner-derived STL model used as the reference standard. Although Primescan has demonstrated high trueness and precision in previous accuracy studies, optical surface scanning is not entirely free of measurement error [24]. Potential sources of uncertainty include scanner trueness and precision, image stitching, the complex peri-implant surface geometry, and the subsequent definition of measurement locations within the STL model. However, the magnitude of measurement deviations reported for Primescan under controlled conditions is substantially smaller than the voxel dimensions used for both CBCT (160 μm isotropic) and PCCT (160 × 160 × 200 μm) in the present study. Therefore, despite its inherent measurement uncertainty, the IOS-derived STL model can be considered an appropriate high-resolution reference standard for the comparison of both radiological imaging modalities. Furthermore, as the same reference dataset and corresponding measurement locations were used for both CBCT and PCCT, any residual uncertainty of the IOS-based measurements would be expected to affect both modalities similarly. Nevertheless, such uncertainty may have influenced the absolute magnitude of the reported measurement errors. Furthermore, only titanium implants were evaluated in this study, which currently still represent the majority of clinically placed implants. However, the imaging characteristics of zirconia implants may differ from the present findings and should therefore be interpreted with caution. Importantly, the imaging systems differ fundamentally in detector technology, acquisition geometry, spectral filtration, tube voltage, and reconstruction algorithms. This technological non-equivalence is inherent to the comparison and represents the core subject of investigation rather than a confounder, as photon-counting CT and cone-beam CT rely on fundamentally different physical principles. However, the individual contribution of specific technical components cannot be isolated.

## Conclusion

Within the limitations of this ex vivo model, PCCT provided more accurate and reproducible measurements of standardized peri-implant bone defects than CBCT at comparable radiation dose levels, confirming the initial hypothesis of this study. These findings demonstrate the diagnostic potential of PCCT for the visualization of peri-implant bone defects. It may potentially replace CBCT one day, offering more reliable three-dimensional imaging while potentially allowing for a further reduction in radiation exposure. However, further in vivo studies including a broader range of clinically representative defect morphologies are required before conclusions regarding clinical implementation or a potential role of PCCT as an alternative to CBCT can be drawn.

## Materials and methods

### Study protocol

**Fig. 3:**
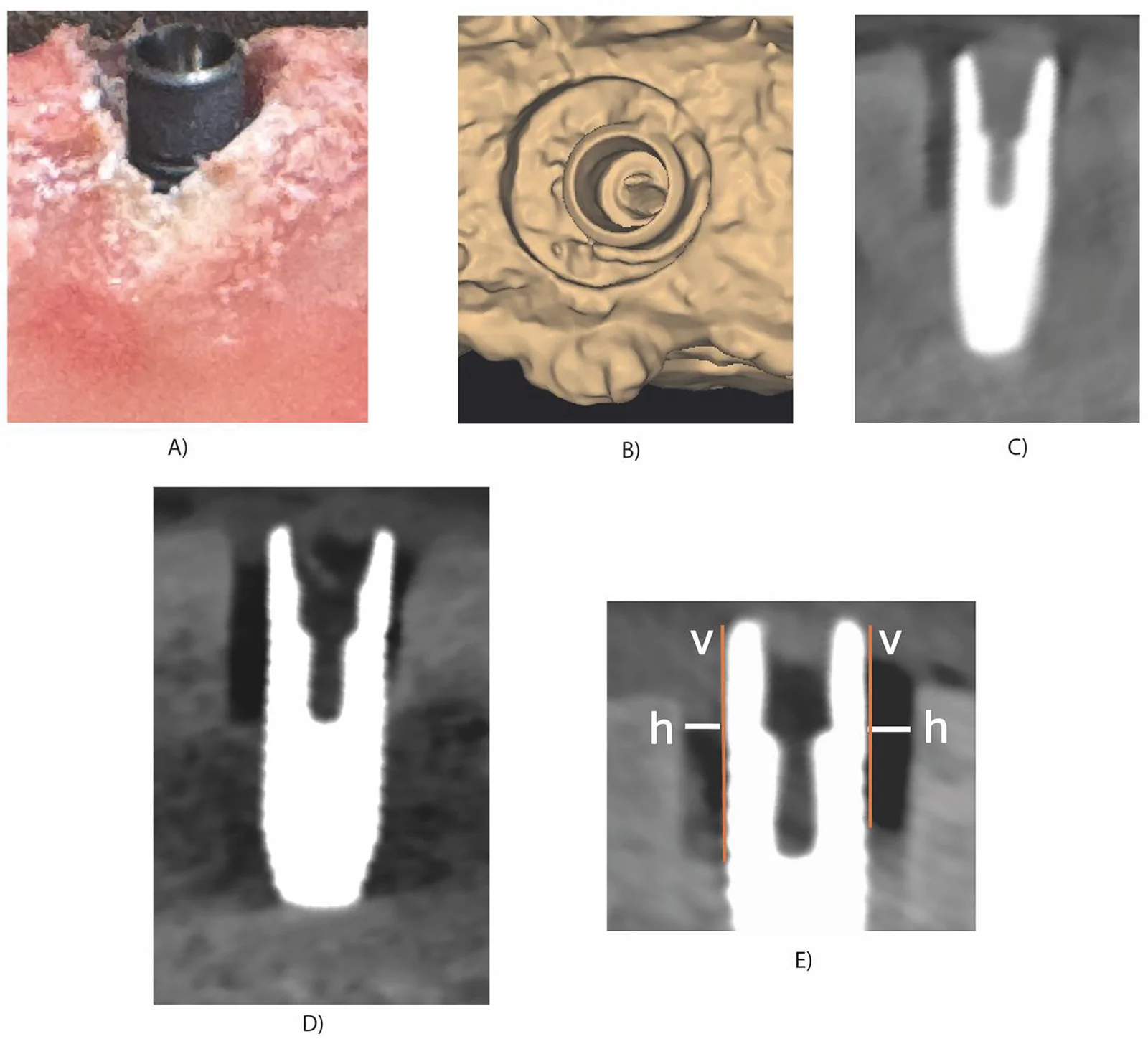
Workflow, imaging modalities and measurement systematics. **(A)** Clinical photograph showing the created peri-implant defect. **(B)** STL scan used as the reference standard. **(C)** Cone-beam computed tomography (CBCT) image and **(D)** photon-counting computed tomography (PCCT) image of the same implant (sagittal section, WL:1024, WW 4096). **(E)** Measurement scheme of the horizontal (h) and vertical (v) defect components. In (**C**) and (**D**), the PCCT image demonstrates superior visualization. In particular, the narrow peri-implant defect on the right side of the implant is clearly delineated in the PCCT image, whereas it appears less distinctly defined in the CBCT image

In total, peri-implant bone defects around 27 titanium implants (Straight Astra Tech Implant System^®^ OsseoSpeed^®^ EV implants, DentsplySirona, Bensheim, Germany) with a diameter of 4.2 mm and a length of 13 mm are examined. These implants are placed in the mandibular angle of 9 porcine mandibles. Initially, the implant sites are prepared in a standardized manner after reflecting the soft tissue with the cortical drilling protocol as suggested by the manufacturer [25]. Following this, the implants are inserted at the crestal level. Subsequently, circular peri-implant defects are artificially created using trephine drills with a larger diameter than the implants. In the process, portions of the vestibular and oral bone are also partly removed to achieve defect morphologies that closely resemble real clinical conditions (Fig. 3A) [11]. Subsequently, the artificially created defects are scanned using an intraoral scanner (Primescan, Dentsply Sirona, Bensheim, Germany) to obtain images that serve as the reference standard (Fig. 3B). The soft tissue is then sutured at the wound margins, ensuring complete coverage of the implants. Subsequently, one high-resolution (HD)-CBCT scan (Orthophos SL 3D, Dentsply Sirona, Bensheim, Germany), commonly used in clinical practice for the visualization of peri-implant tissues, is performed, followed by a photon-counting computed tomography (Naeotom Alpha, Siemens Healthineers, Forchheim, Germany) acquisition of the mandibles (Fig. 3C + D).

### CT systems, image acquisition and reconstruction

The technical parameters are similar to a previous study [18]. Briefly, the CBCT imaging system in this study is an energy-integrating digital volume tomography system (Orthophos SL 3D, Dentsply Sirona, Bensheim, Germany). The system is operated in high-resolution mode using a tube voltage of 85 kV, a tube current of 6 mA, a scan time of 14.2 s and a field of view of 8 × 8 cm^2^ resulting in a dose of 4 mGy (CTDI_16 cm_). Image reconstruction is performed onto an appropriately sized matrix with an isotropic voxel size of 160 μm [11]. A summary of all acquisition and reconstruction parameters can be found in Table 1. The clinical photon-counting CT system (Naeotom Alpha, Siemens Healthineers, Forchheim, Germany) used in this study is a dual-source CT system and provides a field of measurement (FOM) of 50 cm. The photon-counting detector used in each imaging chain can be operated in several distinct modes. A mode referred to as ultrahigh resolution (UHR) is of particular interest herein. Using this mode, the system provides a detector pixel size of 150 μm × 170 μm in the center of rotation and allows for a spatial resolution of up to 42.9 lp/cm (MTF_10%_) [21, 26]. All acquisitions presented herein were performed with the detector operated in UHR mode and using a tube voltage of 140 kV, as well as an additional tin filter. Such tin prefiltration can only be applied in clinical CT systems, as the X-ray tubes in typical CBCT devices are not sufficiently powerful to compensate for the associated photon loss. The tin filter results in additional spectral hardening, thereby improving the penetration of metallic structures and reducing beam hardening artifacts [27]. The tube current was selected to achieve a dose similar to the CBCT acquisitions, resulting in 4 mGy (CTDI₁₆ _cm_). Image reconstruction was performed with an axial voxel size of 160 μm and a longitudinal voxel size of 200 μm using a sharp B72 kernel (15.7 lp/cm MTF_10%_) and the system’s default reconstruction method, i.e., Quantum Iterative Reconstruction (QIR), at a strength level of 3 [13, 14] (Table 1).

**Table 1:** Summary of all scan and reconstruction parameters for the experiments presented herein

	CBCT	PCCT
Dose	0.94 Gy·cm² (DAP) (about 4mGy CTDI_16 cm_)	4 mGy (CTDI_16 cm_)
Tube Voltage	85 kV	140 kV
Additional Prefilter	-	0.4 mm Sn
Trajectory	Circle	Spiral
Reconstruction	FBP	QIR3
Voxel Size (Axial)	160 μm	160 μm
Voxel Size (Longitudinal)	160 μm	200 μm

FBP-filtered backprojection, QIR3-quantum iterative reconstruction with strength 3, DAP-Dose area product

### Image analysis

The vertical defect depth (v) and the horizontal defect width (h) at 2 predefined locations per implant, if possible, were determined in both CBCT and PCCT and subsequently compared to the afore-mentioned reference standard obtained with an intra-oral scanner (Fig. 3E). For measurement and determination of the reference standard, the resulting models in stereolitography (STL) format were imported into appropriate software (Exocad, exocad GmbH, Darmstadt, Germany) and assessed by two experienced readers in consensus (MR and AF). Measurements were performed at one to two randomly selected locations of the defect. Defect depth was measured from the implant shoulder to the base of the defect, and defect width was measured at two predefined points. To enable assessment of the identical locations in CBCT and PCCT datasets, the scans were aligned in a multiplanar reformation view to generate a sagittal 2D slice corresponding exactly to the locations defined in the reference standard. In this image, the sites designated for defect width measurements were marked accordingly. The software OsiriX (Pixmeo SARL, Bernex, Switzerland) was used for the subsequent measurements, which were performed by two calibrated readers (MR (reader 1) and AF (reader 2)) experienced in 3D dental diagnostics. To assess both inter-reader and intra-reader agreement, reader 1 performed the measurements twice with an interval of three months in order to minimize potential recall bias. All measurements were conducted blinded to the reference standard as well as to the previously obtained measurements of the same reader and those of the second reader. Calibration was conducted by having the reader independently evaluate 10 images that were not included in the study, followed by a consensus assessment. If no consensus could be reached, the images were reviewed with a third experienced reader (CM) until agreement was achieved.

### Statistical evaluation

For method comparison analyses, a single representative value per specimen and modality was calculated as the mean of all available measurements (reader 1 repetition 1, reader 1 repetition 2, and reader 2). This approach was chosen to reduce random measurement variability and to provide a stable modality-specific estimate for comparison with the reference standard. Intra-reader reliability was assessed using the two repeated measurements of reader (1) Inter-reader reliability was evaluated by comparing the mean value of reader 1 (average of the two repetitions) with the single measurement of reader (2) Statistical analyses were performed using R (R Foundation for Statistical Computing, Vienna, Austria). Agreement between each imaging modality and the reference standard was evaluated using paired t-tests. When appropriate, Wilcoxon signed-rank tests were additionally performed as robustness checks. Normality of paired differences was assessed using the Shapiro-Wilk test.

Agreement of CBCT and PCCT, respectively, to the reference standard was further characterized using Bland-Altman analysis, including calculation of the mean difference and the 95% limits of agreement. Measurement accuracy was quantified using the mean absolute error.

To account for potential scaling effects due to differences in feature magnitude, additional analyses were performed using relative error metrics. Relative error was defined as (method – reference standard) / reference standard, and relative absolute error as |method – reference standard| / reference standard. Percent Bland-Altman plots were generated by expressing the difference between method and reference as a percentage of their mean value. Differences in absolute and relative absolute errors between CBCT and PCCT were compared using paired one-sided t-tests under the hypothesis that PCCT would demonstrate lower measurement error. Intra- and inter-reader reliability were assessed using intraclass correlation coefficients (ICC) calculated with a two-way mixed-effects model and absolute agreement definition. A p-value < 0.05 was considered statistically significant.

## Data Availability

The datasets used and/or analysed during the current study are available from the corresponding author on reasonable request.

## Abbrevations

CBCT: Cone beam computed tomography
CT: Computed tomography
CTDI: CT dose index
FOV: Field of view
ICC: Intra-class correlation coefficient
PCCT: Photon-counting CT
QIR: Quantum Iterative Reconstruction
SD: Standard deviation
UHR: Ultrahigh resolution
FOM: Field of measurement
STL: Stereolitography
MTF: Modulation Transfer Function at 10% contrast
